# Immersive VR for investigating threat avoidance: The VRthreat toolkit for Unity

**DOI:** 10.3758/s13428-023-02241-y

**Published:** 2023-10-04

**Authors:** Jack Brookes, Samson Hall, Sascha Frühholz, Dominik R Bach

**Affiliations:** 1 Max Planck UCL Centre for Computational Psychiatry and Ageing Research and Wellcome Centre for Human Neuroimaging, UCL Queen Square Institute of Neurology, University College London, London, UK; 2 Cognitive and Affective Neuroscience Unit, University of Zurich, Zürich, Switzerland; 3 Department of Psychology, University of Oslo, Oslo, Norway; 4 Hertz Chair for Artificial Intelligence and Neuroscience, Transdisciplinary Research Area “Life and Health”, University of Bonn, Bonn, Germany

**Keywords:** VR, Decision-making, Motor control, Defensive behaviour, Threat avoidance, Research software

## Abstract

All animals have to respond to immediate threats in order to survive. In non-human animals, a diversity of sophisticated behaviours has been observed, but research in humans is hampered by ethical considerations. Here, we present a novel immersive VR toolkit for the Unity engine that allows assessing threat-related behaviour in single, semi-interactive, and semi-realistic threat encounters. The toolkit contains a suite of fully modelled naturalistic environments, interactive objects, animated threats, and scripted systems. These are arranged together by the researcher as a means of creating an experimental manipulation, to form a series of independent “episodes” in immersive VR. Several specifically designed tools aid the design of these episodes, including a system to allow for pre-sequencing the movement plans of animal threats. Episodes can be built with the assets included in the toolkit, but also easily extended with custom scripts, threats, and environments if required. During the experiments, the software stores behavioural, movement, and eye tracking data. With this software, we aim to facilitate the use of immersive VR in human threat avoidance research and thus to close a gap in the understanding of human behaviour under threat.

## Introduction

The ability to avoid threats is crucial for survival. Biological organisms possess countless mechanisms to this end, from the camouflaging sand-coloured fur of the Sahara-dwelling fennec fox, to the viscus slime produced by glands of the threatened hagfish. However, perhaps the most fundamental survival techniques arise from the basic ability of movement. When faced with a predator, rival individual, or environmental threat, animals exhibit various defensive behaviours, from simple “fight or flight” actions (Cannon, 1915) to sophisticated techniques to ward off opponents such as thanatosis (playing dead), freezing, distraction, or startling (Evans et al., 2019). The high stakes and rapid occurrence of many threat encounters necessitate both speed and precision in the cognitive and neural controllers for action selection and execution (Bach & Dayan, 2017). To date, investigations into the nature of these controllers have largely been limited to non-human species or clinical observations in anxiety disorders (Blanchard et al., 2011; Graeff, 1994; Gray & McNaughton, 2003; Panksepp, 2005; Roelofs, 2017). In contrast, investigating threat avoidance in healthy humans is made impractical by ethical concerns. Here, research has been carried out using techniques of imagined scenarios (Blanchard et al., 2001), capturing metacognitive reflections about how participants believe they would act, or third-person view computer games (Bach, 2022) which allow only for very restricted movements of the fingers or hands that control peripheral input devices, usually keyboard or joystick. Investigating mechanisms that drive realistic action-selection behaviour under threat could allow for a better understanding of anxiety disorders, and inform the development of safety-critical artificial intelligence systems.

VR technology has long been posited as a useful tool for behavioural research, as it combines ecological validity with experimental control (Loomis et al., 1999), and with current technological developments is becoming a promising research tool (Parsons, 2015). Modern VR systems support full 6-degrees-of-freedom movement, meaning that users not only can passively observe, but can actively interact with and naturally traverse, a virtual environment. Full body movement is now also a possibility, since contemporary head-mounted displays (HMD) are lightweight, wireless, and support the use of hands or other body parts to interact with virtual worlds. Moreover, whilst nowhere close to “photo-realism”, current levels of graphics computing power allow for rich three-dimensional (3D) environments to be rendered at high resolutions and frame rates. For just a few examples, recent work has used VR to investigate visually evoked threat responses, conditioned and unconditioned autonomic responses (Rosén et al., 2019), neural mechanisms of context conditioning (Alvarez et al., 2008; Xia et al., 2023), risk assessment strategies, arousal and neural responses during exposure to height (Baker et al., 2020; Krupić et al., 2021; Yilmaz Balban et al., 2021), and biofeedback during police officer training (Brammer et al., 2021). These examples taken together point to an opportunity to address the present gap in human research by using VR technology to study action selection under threat.

Here we present a software toolkit for building experiments to investigate human behaviour under threat. Real-time 3D application development is resource intensive, with game development studios often employing hundreds of artists, animators, programmers, and testers. However, we aimed to satisfy a very specific need, and limited the number of assets. Thus, the project consists of several 3D animated threats, environments, and interaction mechanisms, which can be modified or expanded with commercially available and custom assets. Overall, we set out to allow for rapid development and deployment of novel experimental paradigms involving interaction with threats in VR. Below we outline the technical and practical hurdles that our software overcomes. The toolkit itself is available under an academic license agreement on XIP (https://xip.uclb.com/product/vrthreat-toolkit-for-unity), while example experiments, data and analysis code can be found on the Open Science Framework (OSF) (https://osf.io/2b3k7/). An R package to process output of these games is available on GitHub (https://github.com/bachlab/vrthreat). We have recently used the toolkit to investigate the diversity of human escape patterns, and the computational mechanisms that control these (Sporrer et al., 2022).

## The toolkit

### Overview

The VRthreat Toolkit allows researchers to create and run behavioural experiments that probe human decision-making, action-selection, and motor behaviour under simulated acute threat. The software is built on the Unity engine. It consists of a set of individual software components that can be used and customised by the researcher in order to produce the desired experimental conditions. The software can then be built (compiled) such that the software can be run independently of the Unity Editor for data collection. The basic building blocks of the experiments created with the toolkit are short semi-interactive “episodes” (Table 1), in which a participant may encounter one (or several) animal or environmental threats. The researcher can design episodes such that they place the participant in unique circumstances, making use of obstacles, forageable fruiting plants, and safety areas. The software supports the collection of a plethora of data types during these episodes, including specified behavioural responses such as fruit picking, continuous movement of head and peripheral trackers, and eye tracking data, usually at the frame rate of the VR presentation.

**Table 1:** Glossary of terms used within the toolkit

Term	Definition
Threat	A specific threatening 3D entity or event which the participant encounters within a scenario, in order to elicit actions from the participant. Threat objects are usually programmed to lead to virtual death when touched.
Environment	A naturalistic virtual 3D world with (e.g.,) trees, mountains, rocks, and certain lighting conditions. The objects in the environment would usually be far from the participant and non-interactive. An environment is called a *Location* in the Unity project and is defined in the form of a Unity *Scene*.
Scenario	A predefined set of objects and events, including threat(s), an incidental foraging task, safe area, or objects obscuring view and/or movement. A time limit can be put in place. Scenarios are saved as *Prefabs* within the Unity project.
Episode	An instance of a Scenario combined with a particular Environment. This distinction exists such that the same Scenario can be used across multiple Environments.
Trial	A single instance or “run” of an Episode with a participant. Episodes can be run multiple times, each constituting an individual trial.
Transition environment	A specific featureless environment that the participant is placed in between trials, to allow them to move between their current position and the required starting position for the following trial, and potentially to respond to questionnaires after a trial.

The primary goal of the toolkit is to facilitate natural interactions in a simple and safe manner, whilst still maintaining experimental control. In the following, we discuss the technical components of the software in relation to these three basic requirements.

### Experiment structure

The structure of multiple continuous episodes with limited duration strikes a balance between competing goals: on the one hand, immersion and naturalistic appearance, and on the other tight experimental control, which often mandates rather short trials in typical psychological experiments. On one end of the spectrum, it would be possible to create a VR “survival simulator”, in which the participant visits a seemingly perpetual environment, allowing realistic interactions within that world. With this approach, a researcher may be able to observe human behaviour when confronted with wildlife, predators, environmental threats, other humans, and forageable food. While such a simulation may provide value, participants’ behaviour would have a great impact on what they subsequently experience (e.g., they may opt to stay away from an area filled with predators). At the other extreme, brief encounters with fully pre-programmed (movie-like) threats, such as those commonly used in psychological research, may not elicit the full action repertoire that humans would normally have access to. For example, in non-humans, it is well-known that unavoidable threats elicit behaviours that are distinct from those elicited by avoidable threats (e.g., mouse/rat freezing in fear conditioning vs running in active avoidance experiments) (LeDoux et al., 2017). Also, without sufficient time to prepare and implement an action, the outcome of a threat encounter becomes essentially uncontrollable. Participants may adapt quickly to this situation. Therefore, we opted for a task structure that allows for continuous behaviour and at the same time ensures all participants have similar experiences regardless of their earlier choices—allowing researchers to observe behaviour free of many confounds. This temporal structure resembles that of third-person view games used in psychology and neuroscience (Bach et al., 2014; Mobbs et al., 2007) while allowing for realistic experience and action affordances.

**Fig. 1:**
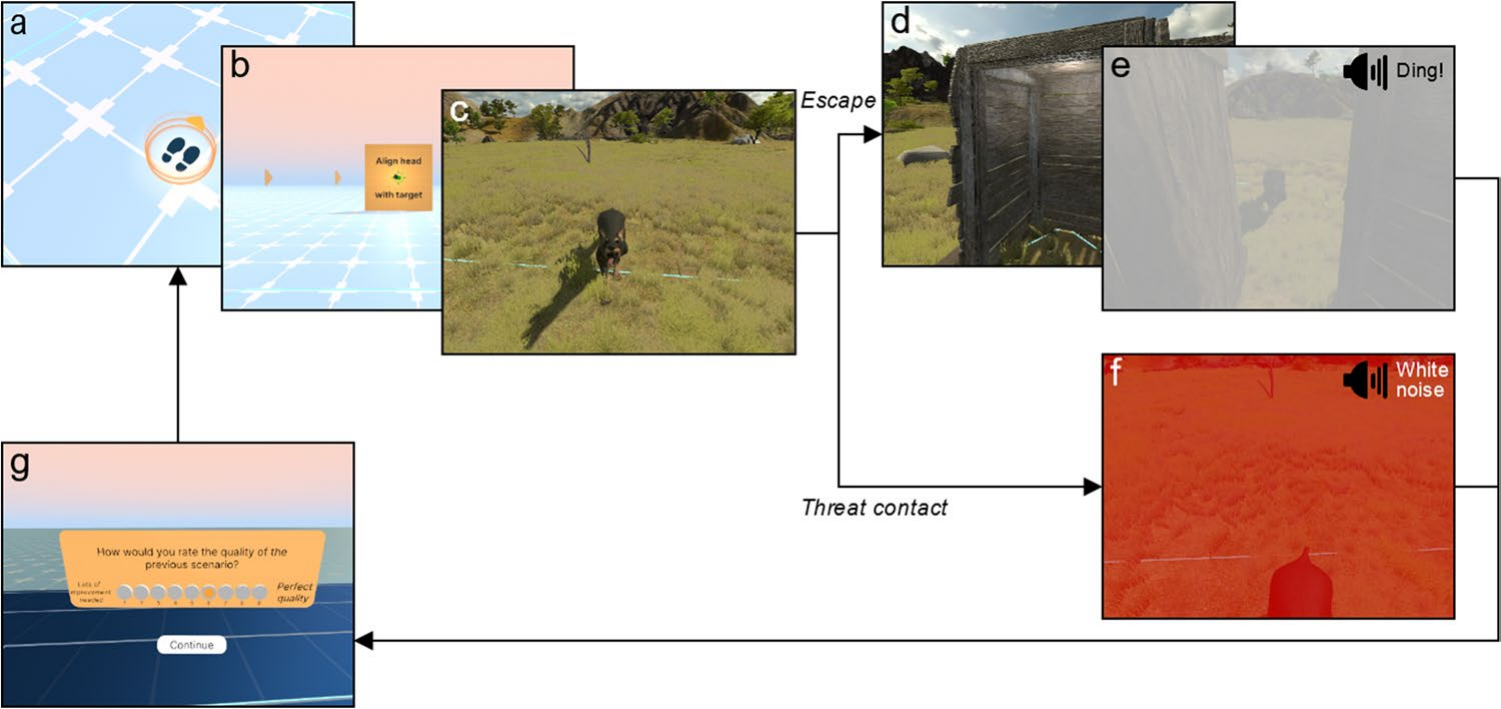
Core experiment loop. (**a**) Participant walks towards a required start location in the Transition environment. (**b**) Participant uses their head to align dot with target. (**c**) The episode begins, here with an aggressive dog running towards the participant. (**d**) The participant may choose to move towards the safety hut. (**e**) Upon entering the safety hut, the door abruptly closes and the screen fades white. Alternatively, if the participant avoids contact with the threat for a set period of time, the screen will also fade to white and the scenario will end. (**f**) If the participant chooses not to escape and instead makes contact with the threat, the screen fades red and a loud white noise sound can be played. (**g**) After (e) or (f), the participant will be brought to an intermediate environment which first displays outcome feedback (e.g., “You survived”) and then may be asked to respond to a question. Then they are put in the Transition Environment and the process repeats, iterating through the trial list as specified by the researcher

Thus, the backbone of the toolkit is a system for building and presenting various “episodes”, i.e., separable, short encounters, with various threats. These episodes are played back in a sequence, with breaks in between (Fig. 1). First, the researcher sets up the participant in the headset, launches the software, and configures the experiment (e.g., entering participant ID, configuring data storage location). Then, the participant will be entered into the transition environment (see Table 1 for a glossary of terms), with only an arrow marker on the floor indicating a place to stand. As soon as the participant stands on this spot, arrows appear around the participant pointing towards a target. The participant aligns their head with the target, ensuring participants are positioned and oriented correctly for the upcoming scenario. The environment and the scenario load, and the participant begins the trial.

Within an episode, we designed several layers of experimental control. The first and simplest layer is the visual and acoustic appearance of the environment, objects, and threats, which we will discuss in conjunction with these assets. The second layer is control over the time point at which a threat is detected, and the spatial position and participant's body posture at this point—which, in non-human animals, is known to affect the available defensive actions (Evans et al., 2019). We primarily sought to design environments in a way that would mandate certain positions and viewpoints, the latter ensuring that a threat would be detected at approximately the same point in time across different participants or trials. To this end, we implemented an incidental task. Since we sought to invoke realism across human history and with no specific cultural associations, the task is to forage for reward-granting fruit. This allows for careful placement of special bushes and trees that direct participants' position, posture, and viewpoint. The third layer is the interaction between threat and participant, which governs to what extent the participant's initial response can lead to deviation between different participants' experiences. The movement of threats (e.g., approaching predator, or rolling boulder) can be specified in advance with temporal and spatial precision. However, whilst identical playback of episodes between participants would allow for precise experimental control, it is likely to bias the participant's actions. For example, they may quickly learn to simply move out of a threat's trajectory, which in reality may not be a predominant action for some threats that chase their prey. Thus, to facilitate natural behaviour and the feeling of immersion, we had to tread a fine line between this precise control and the interactivity of the episodes. This is discussed in conjunction with the threat assets.

The scenario ends at the point at which either (1) the threat “touches” the participant, in which case the screens fade to red and a negative sound effect is played, (2) the participant enters a defined safe area, leading to a fade to white, or (3) the pre-specified time period has elapsed, also fading to white. Then, after a potential short delay (the duration of which can be set to ensure consistent trial duration regardless of outcome), the screen fades back into the intermediate environment. In published games, we have included episodes of around 1–2 minutes maximum duration, but the toolkit allows us to define much shorter or longer episodes as well.

We architected the software such that a list of episodes used for a particular experiment is specified by the researcher in advance; the trial list can be randomly or manually re-ordered if required.

### Art and assets

This platform is intended to be very extensible, and so the full power of the Unity engine is available to the researcher. There are no restrictions on additional assets such as custom scripts, models, and animations. Creating art assets is a resource-intensive task, and so we had to prioritise and create assets that were the most necessary. Several environments, scenario objects, and fully animated threats are included that allow for a wide range of experiments to be built. In general, we set a requirement for assets to be visually plausible in historic and prehistoric times. With respect to human artefacts (e.g., hut, bridge, weapons), we allowed for the appearance of tool use (e.g., smooth rather than raw wood surfaces) but sought to avoid specific culturally relevant objects, or association with specific historical periods or contemporary civilisations.

#### Threats

Threats were selected based on several principles (Table 2). First, we sought to include conspecific, predatory, defensive, disgust-relevant, and inanimate threats. Secondly, we stipulated that threats have considerable prehistoric interaction with humans, which may have led to the evolution of pre-programmed controllers in order to deal with the threat. Next, we prioritised threats that are relevant either in terms of a high number of deaths or injuries caused, or in terms of prevalence in specific phobias. Fourth, the selection of threats was made to cover a wide range of sizes and interaction types, as for example a charging elephant would clearly command different movements from the appearance of a spider close to the hand. Fifth, we considered the existing body of research, and lastly, the implementation difficulty.

**Table 2:** Summary of threat selection principles

Threat selection principles
Variety of threat classes: *conspecific, predatory, defensive, disgust-relevant, inanimate* threats, to account for the possibility of multiple behavioural controllers
Prehistoric presence to account for the possibility of “pre-programmed” controllers arising through natural selection pressures
Relevance: either in terms of significant damage, or in terms of subjective fear ratings or prevalence of phobias
Variety of threat size and attack style that may command a large action repertoire
Existing body of research relating to the neural, physiological, or behavioural response to confrontation with different threats
Implementation difficulty of 3D modelling, texturing, and animation

These considerations led us to develop and include 14 natural threats as well as one artificial threat that participants had never encountered before for comparison (Table 3). We also chose to create an artificial threat which can be modified to serve as a comparison condition in different experiments, for example, investigating whether action selection controllers are activated by the appearance of specific visual features (e.g., sharp teeth). A simple red cube was chosen to fit this need, which can be resized to allow for comparison to different threats, and can be adapted further. The human threats posed a particular challenge, as the “uncanny valley” effect (Mori, 1970) may make interactions feel unnatural. To mitigate this, we added cloth to obscure the faces of the models, also reducing the need for facial animation work.

**Table 3:**
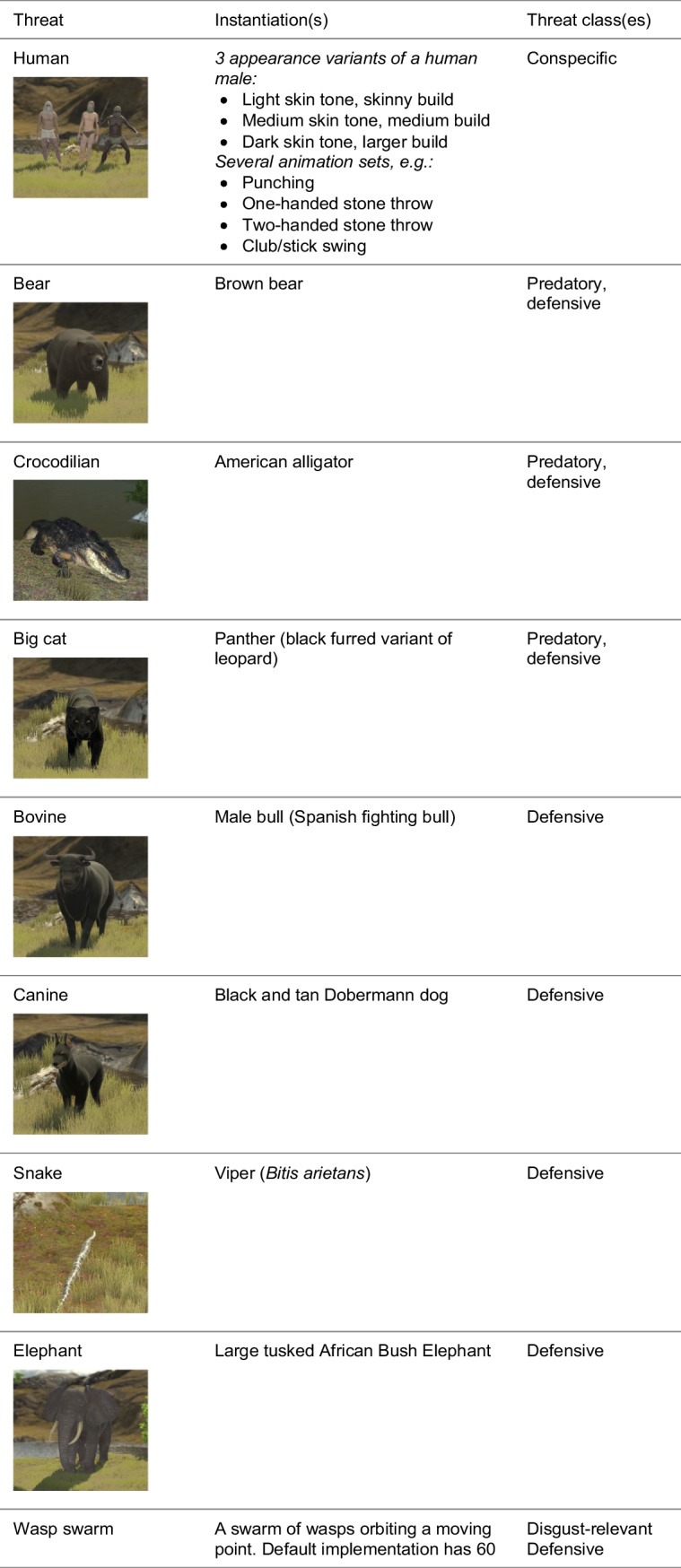

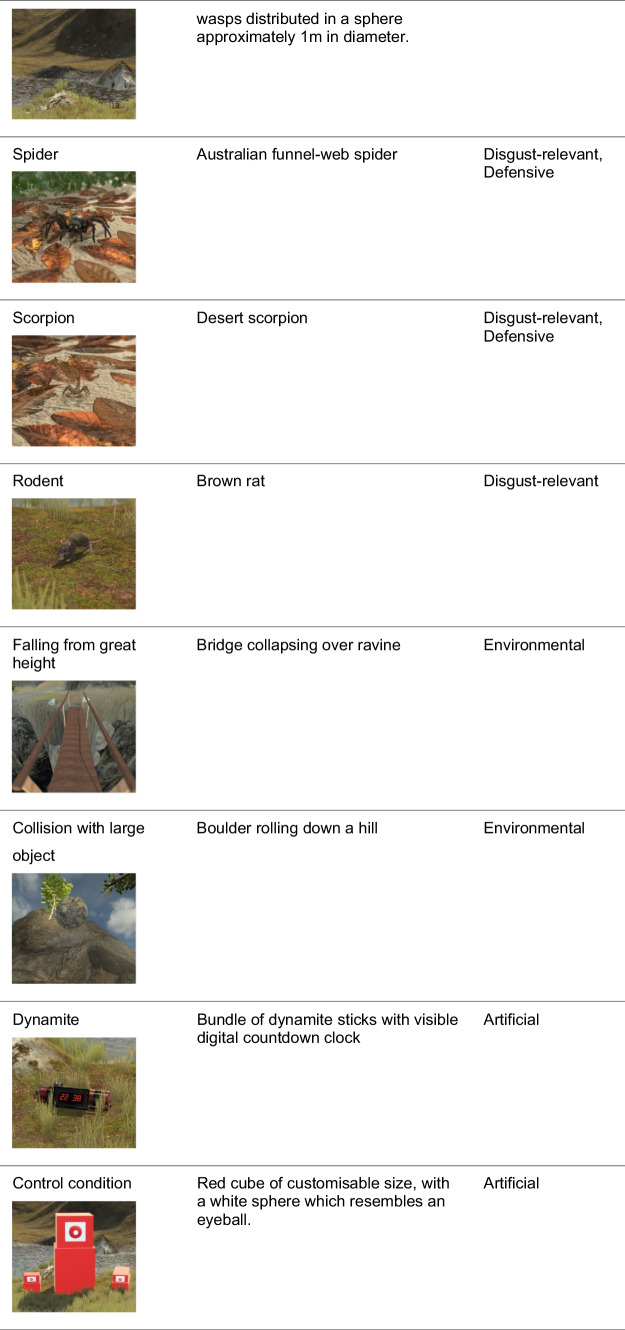
Summar﻿y of threat classes and selected threats. Detailed background information can be found on OSF

#### Threat animations

All threats have a complete suite of animations that allow for a wide variety of scenarios to be created. The human and animal threats are accompanied by animations for locomotion, as well as common behaviours such as grazing. The animations were developed by studying video footage of animal movement, or sourced from online 3D model databases. Unity’s *Mecanim* animation system allows seamless transitions between animations to ensure somewhat realistic movement. Whilst perfect movement is difficult to achieve without hand animation of each scenario, we opted to create just a small number of key animation building blocks, which can be strung together to create a lengthy animation sequence, striking a balance between realism and flexibility. A full list of the included animations is presented in Table 4.

**Table 4:** Core set of animations available across human and animal threat models

Animation	Description
Walk forward	Walking at natural speed for the relevant animal
Turn left/right	Stationary turning pivoting about centre of mass
Idle	Animal resting in natural position, visibly breathing if relevant
Attack	Animal’s attack behaviour, e.g., jumping towards participant
Run forwards	Running at natural medium speed for the relevant animal
Sprint forwards	Running at natural high speed for the relevant animal
Non-threatening behaviour	Animal performing a natural behaviour that it would typically perform when not threatening the participant, e.g., eating grass, sniffing floor, grooming self
Threaten	Display of aggression, e.g., show teeth, widen posture, prick up ears
Blink	Eye blink, speed based on available video footage. Only relevant for animals that blink
Verbalise	Open mouth slightly in relevant animals, accompanied by relevant sound clip

We opted to include some interactive elements to allow for natural interactions and increased immersion. In particular, animal threats can track a participant’s movement, including utilising neck and eye movements, or even fully chasing or following a participant if the participant moves outside of the threat’s planned trajectory. An animal threat can be set to “attack” a participant when at the required distance.

To implement these interactive features, we complemented traditional key-frame animations with procedural (i.e., script-controlled) animation. Working in conjunction with the animations in Table 4, there are two components included which provide this procedural animation for human and animal threats:

- Body Bend system: As a threat moves forward, but the target (e.g., the participant) moves too, the threat must change its path. To do this, the Body Bend system turns joints in the body of the threat to re-orient it. For quadrupeds, this means the front half of the body is turned towards the target, with the back end “snaking” behind it. Parameters of this system can be tweaked to facilitate realistic appearance based on the movement of the threat; these include the turn speed of different joints and the maximum bending angle.
- Head Look system: In pilot testing we found that threats appeared lifeless and still if their eyes and head did not react to changes around them. The Head Look system orients the eyes, head, neck, and potentially upper body, towards a point of interest. It can track an arbitrary point, or also the participant’s head. Parameters here can also be customised to set the turning speed and maximum bending angle of different joints.

#### Sounds

Relevant threat sounds (e.g., animal calls, rock fall, foley) were sourced from various databases due to the technical and practical difficulties involved in recording these sounds. These were temporally matched to threat animation, so that they play back as threats walk or verbalise, for example.

Human vocalisations posed a particular challenge, as we did not want to use words that would be recognisable by participants. Instead, we sought to use outbursts of nonsensical words in differing emotional tones. We selected two nonsensical words [two-syllable words, alternating 5-letter (CVCVC) combinations of consonants (C) and vowels (V)] spoken in angry and happy tones by three male voice actors, similar to those used by Frühholz et al. (2015). These are part of a larger audio pack (ZEMOV: Zurich emotional voices), and so more emotions, words, and speakers can be imported in the future.

#### Environments

We created several environments for scenarios to take place in. These environments are designed to be somewhat realistic settings for encountering threats, and cover a breadth of different levels of visibility, different biomes (grassland, forest, desert), and the topographical layout of the environment (e.g., rocky outcrops or tall grass obscuring the initial view of a threat). Some environments can be used at either daytime or night-time. The included environments are shown in Table 5.

**Table 5:** Included environments. We created a range of different environments of different biomes, time of day, and topography

Environment	Description
Open field	Open grasslands with distant hills and mountains
Cliffside	Open grasslands beside a set of steep cliffs
Ravine	Open grasslands with a large ravine
Savanna	Open desert with gentle slopes and sparse vegetation
Forest	Grassy forest with somewhat tightly packed trees
Swamp	Swamp beside a pool of water, with trees and thick fog
Enclosed mountains	Grasslands surrounded by tall rocky outcrops
Thick grass	Clearing within an area of thick tall grass

Trees, rocks, textures, and water assets are also included for easy development of additional environments. These were sourced from various repositories which allowed re-distribution under their licence agreement.

#### Scenario objects

Aside from threats and environments, additional objects were sourced or developed which will allow us to implement experiments probing specific features of action-selection behaviour. For example:

- **Threat predictors:** Non-human research suggests some actions are not elicited by predictors of threats, but only by the threat itself (e.g., startle reflex: Yeomans et al., 2002). We created several assets that can be used as potential predictors of the presence of a threat. These include natural predictors such as (1) the sound/appearance of a rustling bush, or the vocalisation of an animal; (2) predictors that require some higher-level reasoning such as signposts with warnings or predators; and (3) unnatural predictors that must be learned through association, such as geometric shapes or simple tone sounds.
- **Unnatural action systems**: There are certain actions (such as nose-poking) that animals have difficulty learning to perform in order to avoid threats (Cain & LeDoux, 2007). To investigate whether participants can learn to use particular actions to avoid a threat, we created two assets: (1) a large wire metal frame mesh cage, with a door as an entrance or exit, which can be opened/closed by the participant’s pressing of large buttons on the door frame, and (2) a system which detects a gesture (e.g., the participant raising both hands above their head) and ties that to some event (e.g., the threat stopping their attack).
- **Magical shield:** Some action-selection controllers might be model-based, meaning they utilise an explicit representation of anticipated effects or goals (Proctor & Vu, 2003). In a reinforcer devaluation paradigm, typical actions no longer lead to rewarding states (Dickinson & Balleine, 1994). Whether these actions are then performed or not hints at the nature of the underlying action-selection controller. To assess this in humans, we implemented a protective “shield” in the form of a bubble which surrounds the participant and protects them from any collision with objects. The bubble builds up around the participant with a sound, and then visually disappears, while its protective effect remains. Whether or not the participant’s actions differ with or without the bubble provides insight into the controller driving the selection of actions.
- **Magical force**: Actions performed by participants that lead to valuable states (safety) are more likely to be performed in the future (learning). However, in a threatening situation, if the mechanics of the task are changed such that a typically rewarding action no longer rewards, how easily can humans suppress that action? To address this, we created a “Magical force” system, which detects if the participant is performing a typical action (e.g., escape, or looking at the threat) and immediately kills them, accompanied by a magical sound. This can serve to investigate whether participants can un-learn default actions when they lead to a negative outcome.

#### Facilitating natural action

A crucial requirement for the experiments built with this toolkit is that they must facilitate natural actions. VR allows the creation of experiments with ecological validity whilst maintaining experimental control (Loomis et al., 1999). Modern VR systems also allow for naturalistic actions to be performed. We compiled a list of potential threat avoidance actions that we may expect to observe, using existing research, news reports, wildlife encounter videos, and historic texts. However, due to practical and technical barriers there are still discrepancies in the virtual environment compared to real life. Namely, the experiments will take place in an empty, flat, finite space. This means many haptic cues are not replicated, actions are restricted to a flat plane (e.g., climbing is not possible), and the participant cannot run too far away because of physical walls. Still, we aimed to facilitate natural actions outside of these restrictions by using the following features:

- **General interaction:** Interaction with the virtual world is done in as simple and natural manner as possible. For example, participants never have to press any buttons on the controllers to perform actions. Instead, they just perform actions as they would in real life.
- **Safe hut:** Within the confinement of even a large experimental room, it may not be possible to outrun a threat, so escape may appear pointless to the participant. To avoid this limitation, we created a wooden hut model which can be added to any scenario in order to give participants an obvious escape route. Upon the participant entering the safe area, the door swings shut and the scenario ends. Setting a parameter can delay the ending of the scenario, allowing the participant to stand in the hut for a few seconds, whilst movement and physiological measures are observed.
- **Weapons:** We sought to facilitate actions that participants might spontaneously perform in a truly dangerous scenario. One such action is reaching for improvised weapons (e.g., sticks, stones). Thus, we made it possible to pick up any discovered objects just by hovering the hand on the object. The weapons can be configured to “kill” the threat on contact, ending the scenario. Weapons are to be restricted to particular scenarios in experiments, as we expect a high rate of usage when they are available. We implemented several non-composite weapons as used by non-human primates.
- **Threat interactivity:** The participant has only limited interactivity with the threat, in order to reduce the complexity of scenarios and make episodes more directly comparable between instances. However, we reasoned that some level of interactivity was necessary; otherwise, participants would quickly learn that their actions had no effect. For example, animal threats can be set to chase or follow the participant if they choose to move, by dynamically turning towards the participant’s position. This introduces an issue whereby the threat may unnaturally “ghost” through obstacles whilst chasing. To mitigate this, if a participant hides behind an obstacle (e.g., tree or wall) the scenario will end, and the trial is ended as if they escaped to safety. Additionally, threats can be set to perform actions when at a certain range of the participant whilst chasing, e.g., invoking an attack such as jump and bite.

#### Feedback and reward

Ethical and practical barriers mean that the reward structures of real-life threat encounters are not fully replicated in VR. Failure to avoid a predator, for example, could carry a heavy price in the real world, such as serious injury or death. Humans in hunter–gatherer societies past and present must often weigh up this risk against the need to forage for food. Therefore, we thought it was necessary to replicate this trade-off in our tasks, in an attempt to facilitate similar action-selection processes. To do this, we implemented several features:

- **Fruit-picking incidental task:** In order to give participants an objective, an incidental task can be added into a scenario. This encourages the participant to stand at a certain point and use their hands to collect fruit that appears in front of them. We sought to use a fruit model that is familiar enough to avoid surprise or curiosity in participants. However, well-known fruit may elicit a sense of mismatch when they occur in implausible environments. To circumvent this, we chose a kumquat model. Kumquats look like olive-sized oranges and are thus visually similar to fruits well-known across cultures but do not occur naturally outside Asia such that most participants in the UK or Europe will not have a firm sense of their natural habitat. The fruit appears sequentially and in pseudo-randomised positions on a bush or tree. The incidental task object can be used in four forms, where (1) the fruit appears on a branch (connected to a larger tree) above the participant, (2) the fruit appears on a small tree (~1.5 m height), (3) the fruit appears on a bush atop a flat surface (tree stump), or (4) the fruit appears on a bush on the ground. These four types of tasks make the participant position their body in different configurations, which allows us to indirectly control participants’ viewpoint and posture when the threat appears. To reduce cue conditioning, different coloured variants of the fruit-bearing plant exist. The researcher can tie the monetary compensation of the participant to the number of pieces of fruit collected, in order to motivate exposure to the threat.
- **Feedback:** When the participant is confronted by the threat in the episode, failing to escape or survive long enough, the headset image will quickly fade to red, and optionally, a loud white noise sound will be heard. This stimulus is designed to be physically uncomfortable, trying to evoke the pain one might feel upon being injured by a threat. In addition, participants will get relevant verbal feedback (e.g., “you survived”, “you escaped”, or “you were injured by an elephant”). This can be coupled with an indication of fruit collected in the previous trial as well as the number collected in total thus far. Failure to survive may deduce currently collected fruit from their reward stock, or even remove some previously collected fruit.

#### Experimental manipulation

**Fig. 2:**
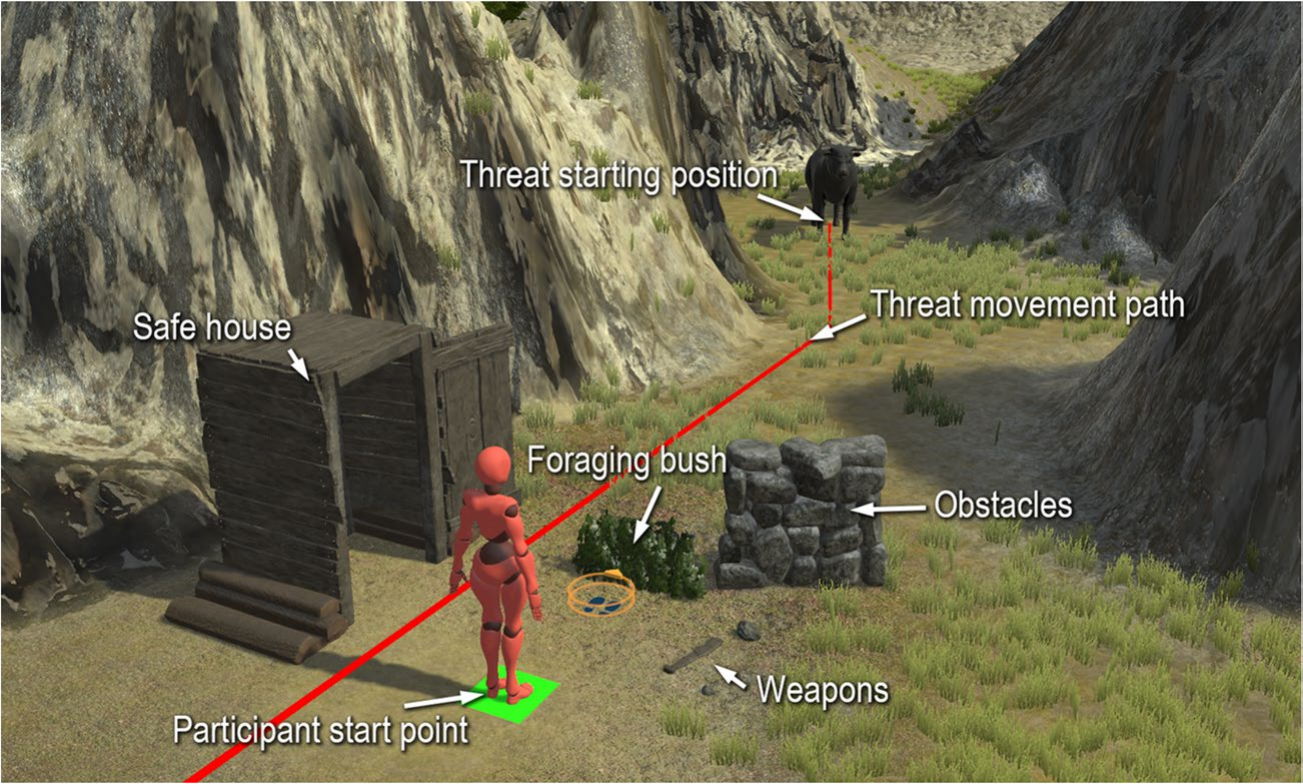
Example scenario built with the scenario builder feature, including a threat movement path defined in the sequence participant. Typical scenario objects are annotated

In order to study the underlying mechanisms of human action selection under threat, we must be able to create experimental manipulations across episodes. By changing aspects of scenarios and measuring the effect on movement, we can gain an understanding of the parameters of action selection in various situations. To facilitate this, a system called the scenario builder is included in the software. It allows objects such as the initial participant position, forageable bushes, safety zones, obstacles, weapons, and initial threat positions to be placed within a scenario (Fig. 2). These are set up to be “drag-and-drop” and require no additional code to be written.

The scenarios we envision building with this toolkit consist of mostly predefined sequences of events, with some small amount of interactivity. The sequence participant component allows researchers to specify the sequence of events that should occur within a scenario with temporal precision. The specification of the sequence is useful for defining the movement of a chosen threat: the researcher can select whether the threat should move towards the participant or an arbitrary point, as well as the speed of movement, among other parameters for fine-tuning the movement. Aside from the movement of the threat, the sequence can include other events, such as playback of a specific threat animation (e.g., a threatening display). The full details of possible sequence events are listed in Table 6.

**Table 6:** Possible Sequence Events used in the Sequence Participant component. The Sequence Participant component allows control of a threat, and other objects in the environment, to create a specific encounter

Event type	Definition
Wait	Simply wait a number of seconds. Can be used to have a threat stay idle for a while between movement.
Rotate towards	Animates the threat such that it rotates its body towards the participant or a point of interest.
Move towards	Animates the threat such that it moves towards the participant or a point of interest. The threat can dynamically alter its path if the target is moving. The acceleration and maximum speed can be set such that the threat either walks or runs.
Animate behaviour	Plays back a specific animation for the threat, e.g., “Threaten”, “Attack” or “Graze”
Head look	Changes the target in the Head Look system to either none (do not move head from default pose), the participant’s head, an arbitrary object, or a random point within an area on the ground in front.
Unity event	Calls any other Unity function the user wishes (e.g., play back a sound of rustling bushes).

These systems work with the Unity prefab system to allow for “variants” of scenarios to be stored in hierarchical fashion. This way, the researcher can create variants (copies of existing scenarios with a small number of changes) where modifications of the existing scenario will propagate to the variant. Typical manipulations made possible by these systems may include:

- Manipulating the presence of threat predictors, such as animal calls or rustling bushes
- Restricting or allowing for certain actions, e.g., removing the safety route, adding obstacles, or scattering weapons on the floor
- Different threats, but keeping all else the same
- Varying the initial threat distance, or distancing based on threat size and run speed
- Varying threat animation, such as grazing behaviour, or threatening display.

#### Data collection features

We implemented several systems to capture various forms of data during experiments.

#### Researcher GUI

**Fig. 3:**
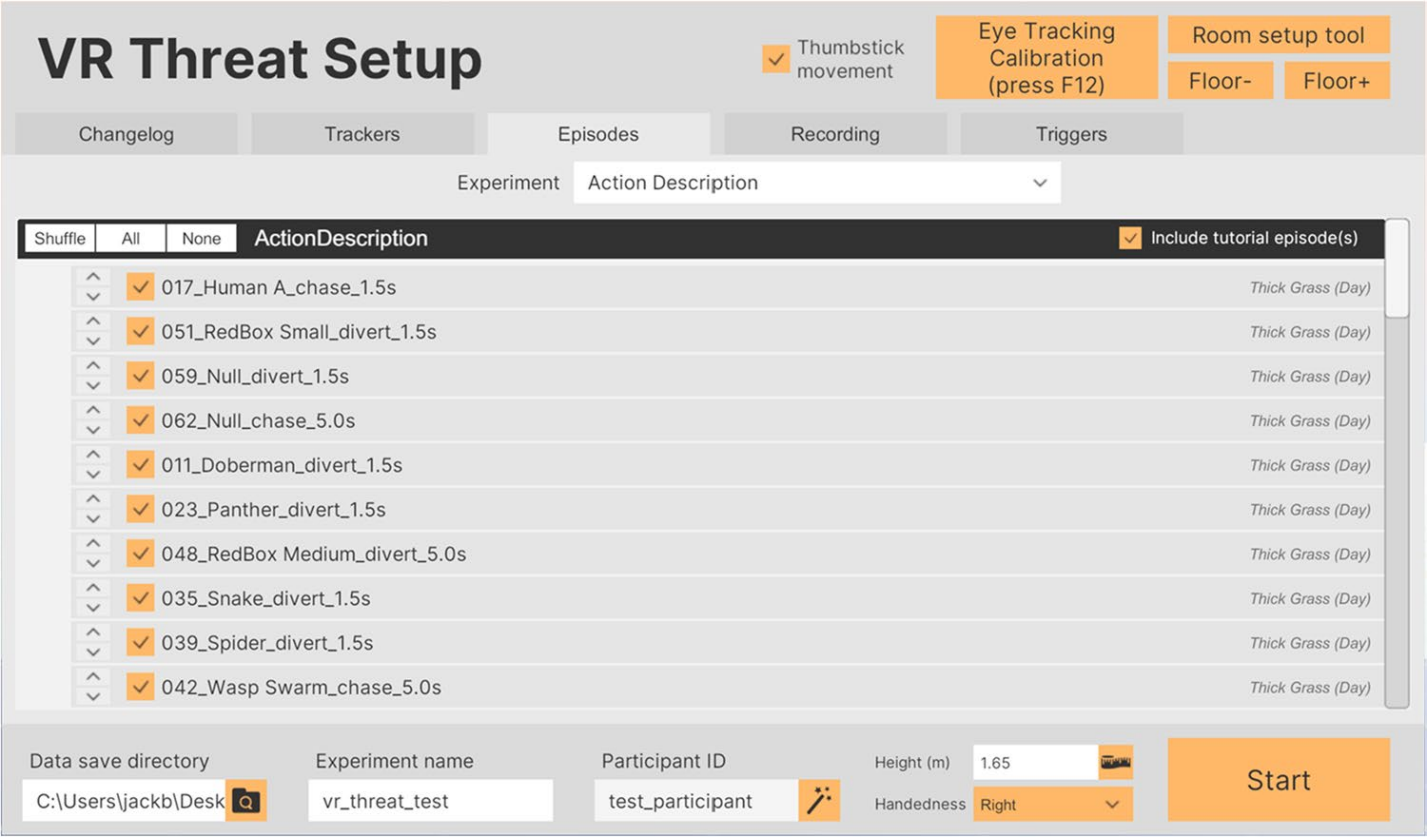
Researcher GUI allowing for selection of data output directory, entry of experiment name and participant ID, participant height and preferred hand, episode selection, and configuring recording options

The toolkit features a GUI (graphical user interface; Fig. 3) that allows researchers to select and order the scenarios required for the experiment, with features like counterbalancing. Additionally, the GUI can be used to configure recording features, such as recording from a microphone, camera(s), and a screen capture of the view from the VR headset.

#### Screen, microphone, and camera recording

The optional screen, microphone, and camera recording is performed by wrapping the popular open-source FFmpeg software (Bellard et al., 2020). The toolkit conveniently splits the recording into files corresponding to trials in the experiment. For microphone recording, modern headsets have a built-in microphone positioned on the underside of the HMD, and will allow any screams or potentially heavy breathing that may occur in response to threatening stimuli to be captured. For the camera recording, the researcher can set up an array of cameras overlooking the physical play space, and recording will be taken from each camera. The videos recorded will be used in conjunction with screen recordings to allow manual investigation of stimuli responses where necessary, but also for 3D markerless pose estimation using software such as DeepLabCut (Nath et al., 2019). For performance reasons, video recording can be done by a second PC; the software sends out recording commands over LabStreamingLayer (Kothe et al., 2012) at the start of each trial. The camera PC listens for these commands, which initiate or terminate recording via the OBS Studio software (Bailey & Contributors, 2017).

#### UXF integration

The toolkit uses Unity Experiment Framework (UXF; Brookes et al., 2019) for trial management and data collection features. This enabled rapid development time, as it avoided the need to manually program this functionality. We use UXF to capture and store as much information as feasible, split across several data types:

- **Behavioural data:** For simple data points such as timestamps when trials start/stop, and the outcome of the episode (confronted threat, escaped, or timed out). These are stored in CSV format with one row per trial.
- **Movement data:** We also log the position/orientation of relevant objects within the VR simulation, these include the hands and head of the participant (as reported by the tracking system of the HMD via Unity), optionally the waist and feet of the participant via VIVE Trackers, and the position/orientation of the threat. These are sampled at the rate that the Unity simulation runs, which is currently tied to the render rate (80–120 Hz, depending on HMD), and are reported in Unity’s coordinate system (1 unit = 1 meter, angles in degrees, left-handed coordinate system). Files are organised with one file per trial.
- **Scenario snapshot output:** To make the experiments as reproducible as possible, a “snapshot” of the scenario is saved on each trial. This is organised as a hierarchical .json file, and stores the name, position and orientation of all objects at the start of that scenario (i.e., initial threat position, position of scenario objects such as blockades or the start point).
- **Subjective response:** As detailed below, the toolkit can query users with a series of questions that appear after a scenario. The data for these questions and responses are stored in a CSV format for each trial.
- **Sequence output:** As described later, a Sequence Participant component within the toolkit allows researchers to specify a predefined set of events that occur in sequence, most notably the movement of a threat. The timings of the sequence are recorded and saved to a readable .json file.

#### Subjective responses

We reasoned that subjective reports would be useful, for example, to assess whether the dimensionality of elicited feelings in real-life scenarios conforms to the circumplex model (Russell, 1980), or for relating subjective response to movements. The participants’ subjective reports are collected using a popup user interface after select scenarios, consisting of a question and a response scale (Fig. 1g). The researcher can select discrete or continuous forms of input for the response. In either case, the participant can simply use their hand to hover over their chosen response or the point on the scale, and confirm their selection. The scales have either customisable icons (e.g., faces depicting an emotional response) or statements, anchored at either end of the scale.

#### Synchronisation with secondary devices

The project is set up to use transistor–transistor logic to send timestamp markers over a parallel port connection to an external device, for example to enable synchronisation with psychophysiological measurement systems including electromyography (EMG), respiratory rate, heart rate, and others.

#### Eye tracking

The project supports the VIVE Pro Eye VR HMD, which includes integrated Tobii eye tracking. The experimenter’s user interface allows easy access to the eye tracking calibration. The eye tracking is currently integrated for data collection only; the software can capture pupil size, eye openness, gaze direction, and an estimate of the object the participant is focusing on using ray casts. These values are provided by the VIVE Eye Tracking SDK (software development kit).

### Technical features

#### Rigidbody recording and playback

The Unity engine includes a physics engine which can be used to simulate the collision of rigid objects. However, due to the discrete nature of the calculations, these simulations can differ between runs even with the same starting conditions. This toolbox includes a recording feature, which will record the trajectories of rigid bodies in a scene (as calculated by Unity’s physics), and allow them to be replayed deterministically in a scenario. The system uses linear interpolation to infer positions from neighbouring data points, since the frame rate of playback can be variable. This system is used for inanimate threats.

#### VR Compatibility

The toolbox interfaces with VR hardware by implementing the SteamVR Plugin, which provides compatibility with all major headsets, but is limited to the Windows operating system. We have tested the toolkit with the HTC VIVE Pro Eye and optional wireless adapter. Episodes can also be tested without a VR headset (controlled using a keyboard and mouse) for rapid prototyping.

#### Audio spatialization

Realistic audio is a significant challenge for VR. We implemented the open-source Resonance Audio plugin for Unity (Resonance Audio, 2020) which simulates spatialization and directionality of audio. This allows sounds such as threat verbalisation to feel like they are positioned in 3D space, in order to increase immersion.

### Health and safety

#### Risk of trauma

The toolkit is designed to place people in threatening scenarios. For ethical reasons, researchers must ensure participants involved in this study do not incur any psychological harm. To do this, we recommend excluding those who have experienced any previous relevant trauma or suffer from any ongoing mental illness. A simple screening tool is the life events checklist LEC-5 (Weathers et al., 2013).

#### Risk of physical accident

The toolkit requires and encourages physical movement around an area in order to interact with the objects in the environment. The urge to escape from a threat may be high—which may increase the risk of participants running into physical walls or other obstacles. We set up a feature of the VR system whereby a bright 3D grid appears, mapping onto the structure of the physical room, when walking close to a wall. We ensure people are familiar with this feature using practice trials.

#### Motion sickness

Many people experience motion sickness in VR, causing short-term discomfort (Davis et al., 2014). The most popular theories posit that feelings of sickness arise from a mismatch of visual and vestibular sensory inputs (Kolasinski, 1995; Sharples et al., 2008). Hence, motion sickness can be reduced by eliminating artificial locomotion, that is, any non-natural translation or rotation of the participant’s viewpoint (Mayor et al., 2021). The toolkit always keeps apparent head motion in line with the participant’s physical movement (i.e., uses purely “room-scale” locomotion), except for a brief moment during “Falling” scenarios, where the participant falls due to gravity during the fade-to-red feedback. We suggest using standardised tests such as the Simulator Sickness Questionnaire (Kennedy et al., 1993) to monitor the impact of motion sickness.

## Discussion

In this technical note, we described the setup of a Unity toolkit for immersive VR research on threat avoidance. Some of the technical features and issues are generic to immersive VR, some others are specific to threat scenarios that mandate rapid spontaneous responses. The toolkit supports simple integration of further threats, non-threat assets, and environments. For example, one could easily include different control conditions without threat, such as commercially available non-attacking domestic animal assets. As another example, if one were interested in application to health and safety research, then it would be simple to add environments and graphical assets that depict typical workplace threats such as a construction environment with scaffolds complemented with falling concrete elements, snapping springs, or trip hazards. In the future, it might be useful to enhance the interactivity of threat assets, in particular if the goal is to investigate defensive behaviour under the perspective of game theory or theory of mind, which might require threat behaviour to change according to the player’s behaviour in more sophisticated ways.

The toolkit contains various assets that are protected by different third-party copyrights. Therefore, access to the Unity project is restricted to academic usage and requires a detailed license agreement, which is available on UCL’s software portal XIP (https://xip.uclb.com/product/vrthreat-toolkit-for-unity). We hope to provide a useful tool for the research community.

## Data Availability

Not applicable

## Code availability

Exemplar games created with this software, as well as examples for output data structure and associated analysis code, are available on OSF (https://osf.io/2b3k7/).

The source code of this software is available under a license agreement (to protect third-party intellectual property) on UCL’s software portal XIP (https://xip.uclb.com/product/vrthreat-toolkit-for-unity).

An R package to process outputs from these games is available on GitHub (https://github.com/bachlab/vrthreat).
